# Transcriptome analysis of the impact of exogenous methyl jasmonate on the opening of sorghum florets

**DOI:** 10.1371/journal.pone.0248962

**Published:** 2021-03-31

**Authors:** Suifei Liu, Yongqi Fu, Yongming He, Xiaochun Zeng

**Affiliations:** 1 Jiangxi Agricultural University, Jiangxi, Nanchang, China; 2 Jiangxi Agricultural Engineering College, Jiangxi, Zhangshu, China; 3 Yichun University, Jiangxi, Yichun, China; South Asian University, INDIA

## Abstract

**Background:**

Methyl Jasmonate (MeJA) could promote the opening of sorghum florets, but the molecular mechanism remains unclear.

**Objective:**

We aimed to investigate the molecular mechanism of exogenous MeJA in promoting the opening of sorghum florets.

**Methods:**

Hybrid sorghum Aikang-8 was selected as the test material in this study. Sorghum plants of uniform growth with approximately 20%-25% florets open were selected and treated with 0, 0.5 and 2.0 mmol/L of MeJA. Totally there were 27 samples with lodicules removed were obtained at different time points and used for the transcriptome analysis using the BGISEQ_500RS platform.

**Results:**

The results showed the sorghum florets opened earlier than the control after the treatment with exogenous MeJA, and the promotive effect increased along with the increase of exogenous MeJA concentration. The number of differentially expressed genes (DEGs) in plasma cells increased with the increase of MeJA concentration, whether up- or down-regulated, after the exogenous MeJA treatment. Besides, the number of metabolic pathways was also positively correlated with the concentration of MeJA. GO and KEGG analysis suggested the DEGs were mainly enriched in starch and sucrose metabolism-related pathways (i.e., LOC8063704, LOC8083539 and LOC8056206), plant hormone signal transduction pathways (i.e., LOC8084842, LOC8072010, and LOC8057408), energy metabolic pathway (i.e., LOC8076139) and the α-linolenic acid metabolic pathway (i.e., LOC8055636, LOC8057399, LOC8063048 and LOC110430730). Functional analysis of target genes showed that two genes named *LOC-1* (LOC8063704) and *LOC-2* (LOC8076139) could induce the earlier flowering of *Arabidopsis thaliana*.

**Conclusion:**

The results of this study suggest that exogenous MeJA treatments could induce the up- or down- regulation of genes related to starch and sucrose metabolism, -linolenic acid metabolism and plant hormone signal transduction pathways in the plasma cells of sorghum florets, thereby promoting the opening of sorghum florets.

## Introduction

In northern regions of China, sorghum was widely planted as a major food crop as well as other important resources for forage, food and brewing industries. So the research of floret opening became increasingly important in sorghum for the growing demand of people. Different species and varieties, and even the flowering time of spikelet at different positions on the same spike in one day are also different, which are affected by the characteristics of varieties and external environment factors [1]. At present, there are many documents on the regulation of spikelets opening influenced by external environment factors, resembling light, temperature, CO_2_ and mechanical stimulation, but the research about the spikelets opening regulated by endogenous signal molecules were still unknown [2–4].

Floret opening in grass plants is mainly caused by the swelling of a pair of lodicules at the base of the floret after water absorption [5]. In this process, the lodicules first swell and push chaff outward and palet inward, leading to the separation of chaff and palet, then, florets open [6]. The normal growth of plants are always regulated by a series of plant hormones, such as Ethylene (ETH), Auxin (IAA), Cytokinin (CTK), Gibberellic acids (GAs), Abscisic Acid (ABA), Jasmonic Acids (Jas), Brassinosteroids (BRs), etc. To date, at least five kinds of plant endogenous hormones can influence the open of plant flowers, including ETH, Jas, IAA, GAs and ABA [7,8]. As a typical C4 plant, sorghum is one of the model plant for the research of comparative genomics of cereal crops [9,10], as well as one of important food crops and energy crops with the greatest potential in China [11]. As it is known to all, the opening peak of sorghum is mainly at night, which is quite different with rice and some other crops. Documents indicated that the opening of floret in excised spikelets in *Sorghum bicolor* L. Moench and *Sorghum Sudanesis* (Piper) Stapf was significantly stimulated by immersing into 2 mM methyl jasmonate (MeJA) solution [12]. So it is quite necessary to research the effect of exogenous methyl jasmonate on the opening of florets in sorghum.

RNA-seq is an very effective tool for studying the regulatory mechanism in plants, such as wheat [13], rice [14], cotton [15]. To date, several transcriptomics studies had been completed on sorghum using RNA-seq method to monitor gene expression or to identify genome-wide SNPs that potentially enhance genetic analysis and the application of molecular markers in sorghum in response to osmotic and abscisic acid and some other biological processes [10,16]. In addition to physiologic or agronomic approaches, RNA-seq could offer new opportunities for dissecting quantitative traits, paving the way to marker–assisted selection (MAS) breeding [17]. In this study, the BGISEQ_500RS sequencing platform was used to compare the transcriptome changes of lodicules of sorghum treated with 0, 0.5 and 2.0 mmol/L MeJA. We focused on the genes and pathways which were closely related to the regulation of floret opening to further elucidate the impact of exogenous jasmonate acids on the floret opening of grass plants.

## Materials and methods

### Materials

In the study, sorghum (*S*. *bicolor* L. Moench) cultivar Aikang-8 was purchased from Shandong Ruiyou Agricultural Science and Technology Development Co., Ltd. in China and used to conduct the experiment. Aikang-8 seeds were planted in the training base of the Jiangxi Agricultural Engineering College On May 1st and May 10^th^ for two times. Each time Aikang-8 was planted for 40 m^2^ with normal cultivation managements. The MeJA (analytical reagent) was purchased from BOMEI (China). It was diluted into 10.0 mmol/L mother liquor with 1.0% ethanol, followed by further dilutions with 1.0% ethanol to 0.5 and 2.0 mmol/L. 1.0% alcohol was used for the blank control. *Arabidopsis thaliana* of wild-type was planted in small pots with a uniformly mixed mixture of peat, vermiculite, perlite by the ratio of 2:7:1 in growth chamber, respectively. The humidity was set at 60% and the temperature was set at 20–22°C. The photoperiod was 24h and the illumination intensity was set 80–200 μmol·M^-2^·S^-1^. Agrobacterium-mediated genetic transformation method was used to infect the inflorescence of *Arabidopsis thaliana* for 2–3 times. Arabidopsis plants were moved to dark environment for 16-24h after the infection. Arabidopsis seeds were harvested and used for the screening of transgenic plants.

### MeJA treatments and sampling

In total, 10 robust and uniform sorghum plants with similar floret opening states were selected and treated with 20 mL of 0, 0.5 and 2.0 mmol/L MeJA, and the plants were then covered with green plastic film. Treatments were conducted at 18:00 PM. The duration from the beginning of treatment to the opening of a large quantity of florets, the number of sorghum plants with a large quantity of florets opened and the numbers of opened florets were recorded. The experiment was repeated three times.

After the treatments, nine samples were collected: samples treated with 2.0 mmol/L MeJA at 19:00 (1h) and 20:30 (2.5h) were labeled as SorgHM_1 and SorgHM_2, respectively; samples treated with 0.5 mmol/L MeJA at 19:00 (1h), 20:30 (2.5h) and 22:30 (4.5h) were labeled as SorgLM_1, SorgLM_2 and SorgLM_3, respectively; and samples treated with 0 mmol/L MeJA at 19:00 (1h), 20:30 (2.5h), 22:30 (2.5h) and 0:30 (6.5h) were labeled as SorgCK_1, SorgCK_2, SorgCK_3 and SorgCK_4, respectively. Each sample was repeated three times. Therefore, there were 27 samples were obtained with lodicules removed, followed by the storage in cold-proof RNase-free tubes at -80°C. The weight of each sample was greater than 0.13 g.

### Total RNA extraction, library construction and transcriptome sequencing of sorghum lodicules

Total RNAs of lodicules were extracted using pBIOZOL method, and RNA concentrations were measured using Nanodrop 2000, followed by RNA integrity test using 1.0% agarose gels. Agilent 2100 Bioanalyzer (Agilent RNA 6000 Nano Kit) was used to detect the concentrations, RIN values, 28S/18S values and fragment sizes of the total RNAs. The results indicated that 27 samples were qualified for library construction. The same amount of RNA from each of the three replicates at each time point were collected and uniformly mixed into nine samples for the sequencing and cDNA library construction. Nine libraries were named libSorgHM_1, libSorgHM_2, libSorgLM_1, libSorgLM_2, libSorgLM_3, libSorgCK_1, libSorgCK_2, libSorgCK_3 and libSorgCK_4, respectively. Library was constructed as follow procedures: Magnetic beads with oligo (dTs) were used to enrich for mRNAs having polyA tails. A DNA probe was hybridized with the rRNA, and RNaseH was used to selectively digest DNA/RNA hybrids. Then, the DNA probe was digested using DNaseI. Target RNA was obtained through purification, and interruption buffer was used to obtain RNA fragments. This was followed by reverse transcription using a random N6 primer. Double-stranded DNA was obtained through the synthesis of double-stranded cDNA. The 5’-end of the synthesized double-stranded DNA was phosphorylated, with an “A” tail at its 3’ end. A connector with a “T” bulge at its 3’ end was ligated as well. The ligated product was PCR amplified using specific primers. The PCR product was heated and denatured into single strands. The obtained single-stranded DNA was circularized using a bridge primer to obtain a single-stranded circular DNA library for further sequencing. Library construction and sequencing were performed by BGI, China.

### Data quality control, filtration and comparison

The filtration of raw reads was performed using SOAPnuke (v1.5.2) [18] by BGI, including the removal of joint reads (joint contamination), the removal of reads with unknown bases at a proportion greater than 10% and the removal of low quality reads (reads with mass values of less than 15 are low quality reads, that accounted for more than 50% of the total bases). The clean reads after filtration (clean reads) were saved in FASTQ format [19]. Hierarchical indexing for spliced alignment of transcripts (HISAT, v2.0.4) [20] was used to blast the clean reads to a reference genome (sorghum genome) and reference gene sequences (sorghum reference genome database information was from https://www.ncbi.nSorgLM.nih.gov/genome/?term=Sorghum%20bicolor%20) [21]. The Mapping rate was calculated using bowtie2 (v2.2.5), and the relative abundances of transcripts were quantified by the RSEM package [22,23]. The average comparison rate of each sample reached 95.39%, and the uniform comparison rate among samples indicated that the data were comparable. Only the clean reads which were successfully mapped on the reference genome were used for subsequent bioinformatics analysis.

### Screening and functional annotation of differentially expressed genes (DEGs)

Clean reads were mapped to the reference sequence using Bowtie2 [24], and gene expression levels were calculated using RNA-seq Expectation–Maximization [25]. The method previously published by Audic and co-workers [26] was used to screen DEGs with the false discovery rates ≤ 0.01 and |log_2_ratio| values ≥ 1. The up- and down- regulation modes of the corresponding samples were described and analyzed. The DEG sequences were mapped to the Kyoto Encyclopedia of Genes and Genomes (KEGG) database using BLAST (parameter: -p blastx-e1e-5-m8) [27]. An enrichment analysis was performed for potential metabolic pathways containing the DEGs. The significant enrichment for a DEG was defined as Q-value ≤ 0.05. The GO framework for model biological systems included three main categories: biological process, cellular component and molecular function [28] and GO terms with a FDR-corrected *p*-value < 0.05 were considered as significant enrichments.

### Verification of DEGs by quantitative real-time fluorescence PCR (qRT-PCR)

The extracted RNAs were used as templates for reverse transcription with a RevertAid First Strand cDNA Synthesis Kit (Thermo Scientific) and several DEGS were randomly selected. The expression levels of genes were verified using SYBR^®^ Select Master Mix (2X) reagents (ABI Inc.) and qRT-PCR. *GAPDH* (AK064960) was selected and used as the reference gene. The relative expression analysis of selected genes was calculated using the 2^−ΔΔCt^ method with triplicate for each sample [29,30].

### Construction of plant overexpression vector

Two target genes *LOC-1* and *LOC-2* were cloned with cDNA by PCR method using 50 μl reaction system, containing 10×PCR Buffer 5 μl, MgCl_2_ (25 mM) 3 μl, dNTP mixture (2.5 mM) 4 μl, forward primer (10 μM) 1 μl, reverse primer (10 μM) 1 μl, Taq (5U/μl) 0.5μl, cDNA 2μl, ddH_2_O 3.5μl. After the purification, PCR products were linked to pMD19-T Vector and transformed into DH5α according to the optimized procedure. At the same time, vector pCAMBIA1300 and pBI121 were digested with EcoRI and HindIII simultaneously. Klenow fragment of pCAMBIA1300 and small fragment of PBI121 were recycled and linked to construct an integrated expression vector pCAMBIA1300-BI containing 35S promoter, GUS gene and terminator. Then the GUS gene was replaced with two target gene *LOC-1* and *LOC-2*, which were added CaMV35s promoter and Nos terminator at 5’ and 3’ end, respectively. The constructed plasmid was transformed into *Arabidopsis thaliana* to verify its functions [31].

## Results

### MeJA can significantly stimulate the opening of sorghum florets

**Fig 1:**
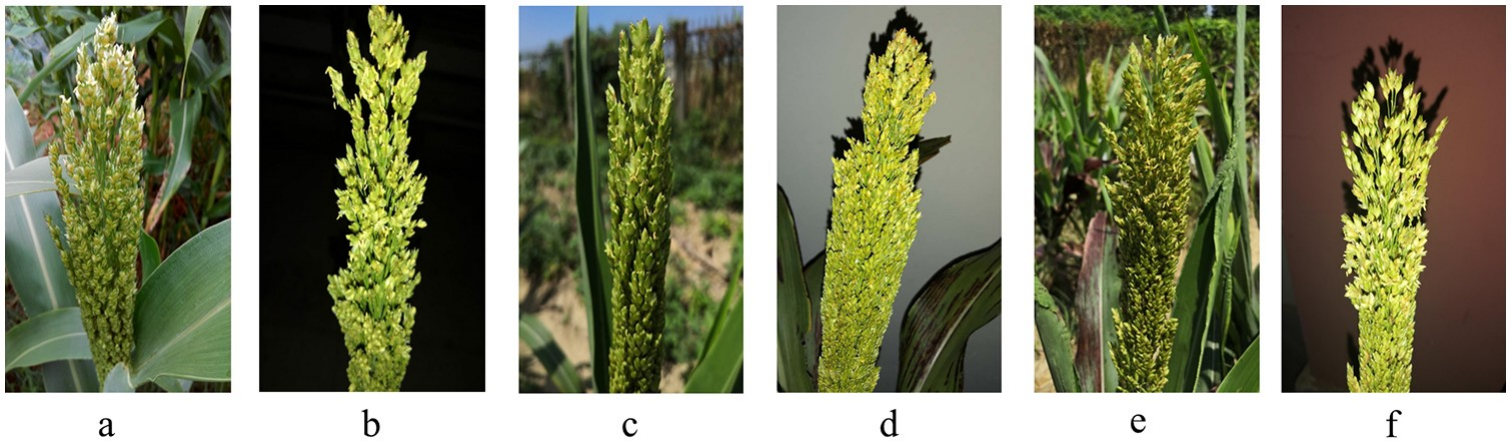
The opening state of sorghum florets after exogenous methyjasmonate treatments. a-0h after the treatment of 0.0 mmol/L, b-6.5h after the treatment of 0.0 mmol/L, c-0h after the treatment of 0.5 mmol/L, d-4.5h after the treatment of 0.5 mmol/L, e-0h after the treatment of 2.0 mmol/L, f-2.5h after the treatment of 2.0 mmol/L.

**Fig 2:**
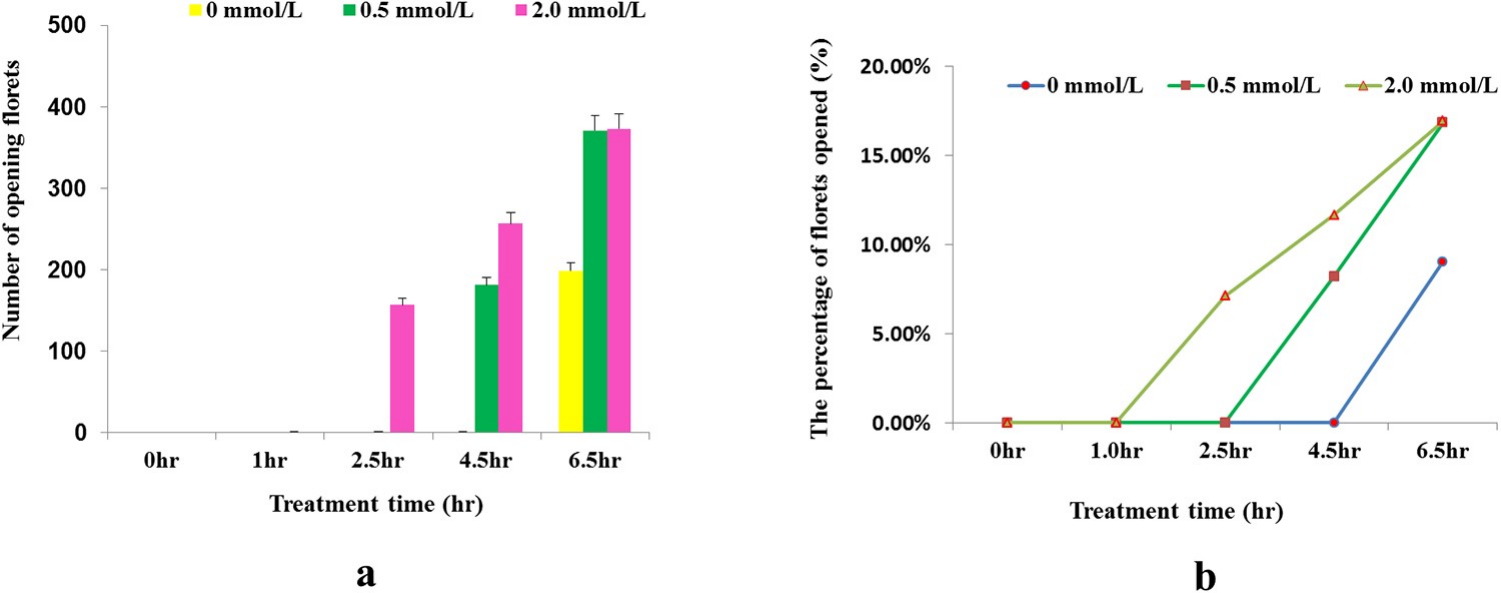
Florets opened in abundance after the treatments. a, The number of florets opened after the treatments. b, The percentage of florets opened after the treatments. In figure a and b, X-axis represents the duration of treatment from 18:00 PM at which time point the treatments were conducted. Y-axis represents the number of opening florets and the percentage of open florets in figure a and b, respectively. After 2.5h of the treatment, the florets began to opening in abundance, and the higher the concentration of MeJA, the more florets opened.

After the treatment at 18:00 PM, the florets did not open until 19:00 after treatment with the three MeJA concentrations. At 20:30 (2.5h), a large number of sorghum florets started to open under the treatment of 2.0 mmol/L MeJA, while other sorghum florets didn’t open in abundance. At 22:30 (4.5h), a large number of sorghum florets started to open under the treatment of 0.5 mmol/L MeJA while the control sorghum florets (0 mmol/L MeJA) still didn’t open in abundance. At 0:30 (6.5h), a large number of control sorghum florets started to open (Fig 1). Besides, the numbers of opening florets after the treatments were also recorded in Fig 2, showing higher concentration of MeJA can obviously induce the opening of sorghum florets in advance. In order to investigate the regulation mechanism in this process, RNA-sequencing work was conducted at each time point.

### Sequencing quality analysis

As shown in Table 1, 27 samples were obtained and sequenced. Finally an average of 24.11 Mb raw read and an average of 24.03 Mb clean reads were obtained. The average total size was 1.2 Gb. Bases with mass values of greater than 20 accounted for 97.70% of the total bases [Clean Reads Q20 (%)]. Bases with mass values of greater than 30 accounted for 90.04% of the total bases [Clean Reads Q30 (%)]. Filtered reads [Clean Reads Ratio (%)] accounted for 99.68% of the total bases. Clean reads which were successfully mapped to the referenced genome (Total Mapping Ratio) accounted for 95.39% of the total reads. Clean reads which were successfully mapped to unique sites on the referenced genome (Unique Mapping Ratio) accounted for 87.74%. In addition, a total of 29,123 genes were detected. All results showed that the data of sequencing quality was qualified for further analysis.

**Table 1:** Read quality levels of 27 samples and comparisons with reference genomes after genomic filtration.

Sample	Total Raw Reads(Mb)	Total Clean Reads(Mb)	Total Clean Bases(Gb)	Clean Reads Q20(%)	Clean Reads Q30(%)	Clean Reads Ratio(%)	Total MappingRatio(%)	Uniquely MappingRatio(%)
SorgCK_1_1	24.14	24.06	1.20	98.06	91.23	99.67	95.50	87.57
SorgCK_1_2	24.14	24.05	1.20	97.86	90.53	99.66	95.62	87.87
SorgCK_1_3	23.87	23.79	1.19	96.55	87.18	99.68	94.94	5.85
SorgCK_2_1	24.14	24.06	1.20	97.76	90.03	99.69	95.46	87.80
SorgCK_2_2	24.14	24.06	1.20	98.01	91.08	99.69	95.32	87.72
SorgCK_2_3	24.14	24.06	1.20	98.00	90.98	99.68	95.63	87.94
SorgCK_3_1	24.14	24.06	1.20	97.89	90.72	99.68	95.78	88.09
SorgCK_3_2	24.14	24.06	1.20	98.00	91.17	99.67	93.92	86.48
SorgCK_3_3	24.06	23.99	1.20	97.49	89.13	99.70	95.30	87.60
SorgCK_4_1	24.05	23.96	1.20	98.10	91.36	99.66	94.69	87.63
SorgCK_4_2	24.06	23.98	1.20	97.93	90.78	99.70	95.33	88.10
SorgCK_4_3	24.05	23.97	1.20	97.98	90.86	99.68	95.31	87.98
SorgHM_1_1	24.14	24.05	1.20	97.88	90.65	99.67	95.50	87.69
SorgHM_1_2	24.14	24.06	1.20	98.02	91.19	99.68	95.27	87.50
SorgHM_1_3	24.14	24.06	1.20	97.78	90.15	99.69	95.55	87.69
SorgHM_2_1	24.08	24.01	1.20	96.65	86.38	99.71	95.49	88.06
SorgHM_2_2	24.14	24.06	1.20	96.98	87.65	99.7	95.37	87.79
SorgHM_2_3	24.14	24.07	1.20	96.12	85.05	99.72	95.72	88.03
SorgLM_1_1	24.14	24.06	1.20	98.08	91.27	99.68	95.36	87.70
SorgLM_1_2	24.14	24.06	1.20	97.78	90.33	99.68	95.09	87.11
SorgLM_1_3	24.14	24.06	1.20	97.86	90.64	99.68	95.81	87.96
SorgLM_2_1	24.14	24.06	1.20	97.9	90.68	99.68	95.80	88.11
SorgLM_2_2	24.14	24.06	1.20	97.97	91.03	99.67	95.34	87.96
SorgLM_2_3	24.14	24.06	1.20	97.74	90.09	99.67	95.76	87.92
SorgLM_3_1	24.04	23.96	1.20	97.93	90.77	99.66	95.24	87.96
SorgLM_3_2	24.14	24.06	1.20	97.75	90.08	99.68	95.71	88.61
SorgLM_3_3	24.14	24.06	1.20	97.70	90.01	99.68	95.79	88.38
Average	24.11	24.03	1.20	97.70	90.04	99.68	95.39	87.74

Sample: Sample name; Total Raw Reads (Mb): Number of reads before filtration; Total Clean Reads (Mb): Number of reads after filtration; Total Clean bases (Gb): Total number of bases after filtration; Clean Reads Q20 (%): Proportion of filtered reads with mass values greater than 20 out of the total number of bases; Clean reads Q30 (%): Proportion of filtered reads with mass values greater than 30 out of the total number of bases; Clean Reads Ratio (%): Proportion of filtered reads ratio; Total Clean Reads: Filtered reads; Total Mapping Ratio: Ratio of clean reads mapped to the reference genome; Unique Mapping Ratio: Ratio of clean reads uniquely mapped to specific sites on the reference genome.

### Analysis of differentially expressed genes (DEGs)

**Fig 3:**
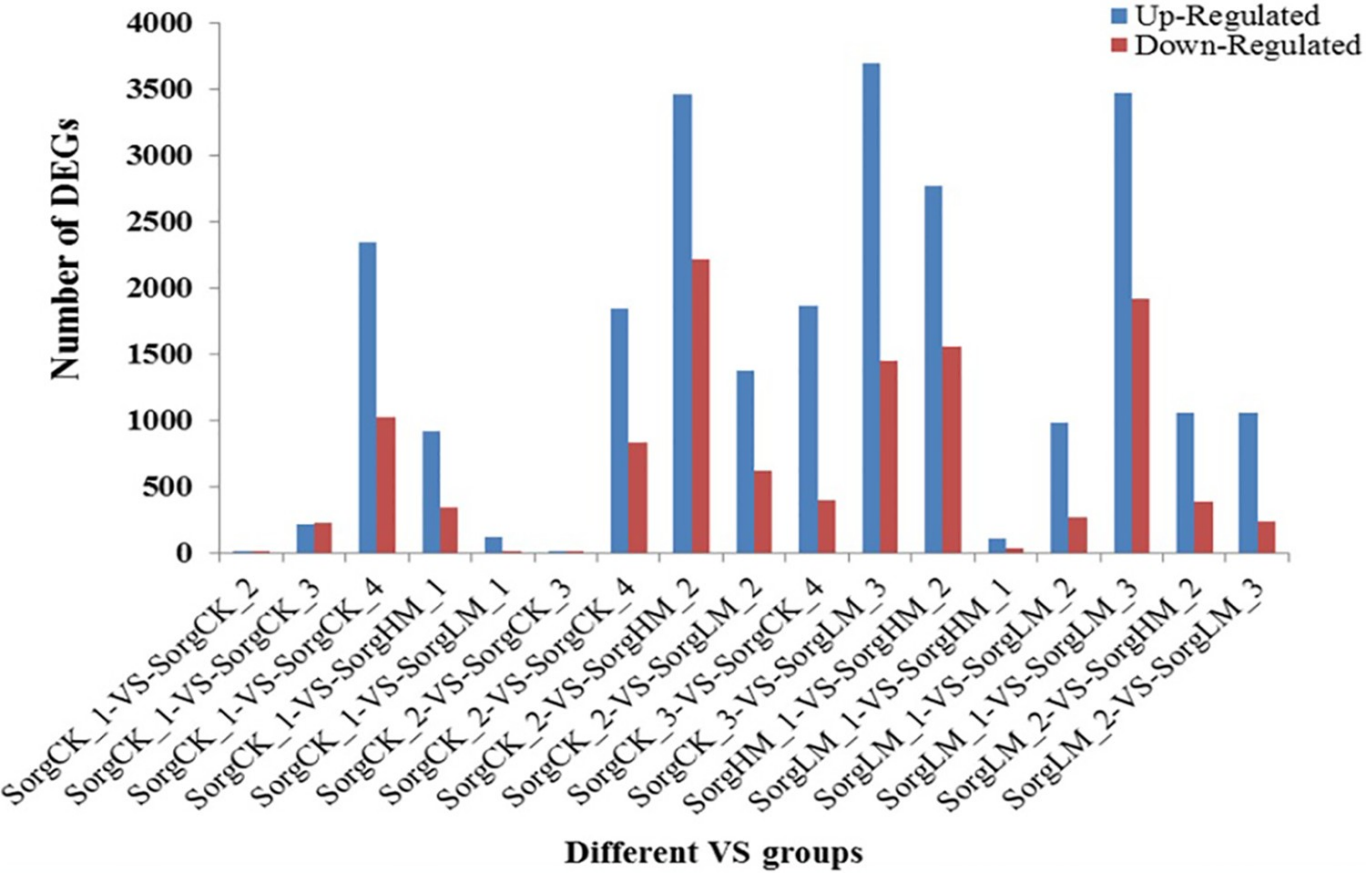
Number of differentially expressed genes (DEGs). X-axis represents different VS groups, and down-regulated and up-regulated DEGS were calculated. Y-axis represents the number of DEGS in each VS groups. SorgCK_1, SorgCK_2, SorgCK_3 and SorgCK_4 represented samples treated with 0 mmol/L MeJA at 19:00 (1h), 20:30 (2.5h), 22:30 (2.5h) and 0:30 (6.5h), respectively. SorgHM_1 and SorgHM_2 represented samples treated with 2.0 mmol/L MeJA at 19:00 (1h) and 20:30 (2.5h), respectively. SorgLM_1, SorgLM_2 and SorgLM_3 represented samples treated with 0.5 mmol/L MeJA at 19:00 (1h), 20:30 (2.5h) and 22:30 (4.5h), respectively.

**Fig 4:**
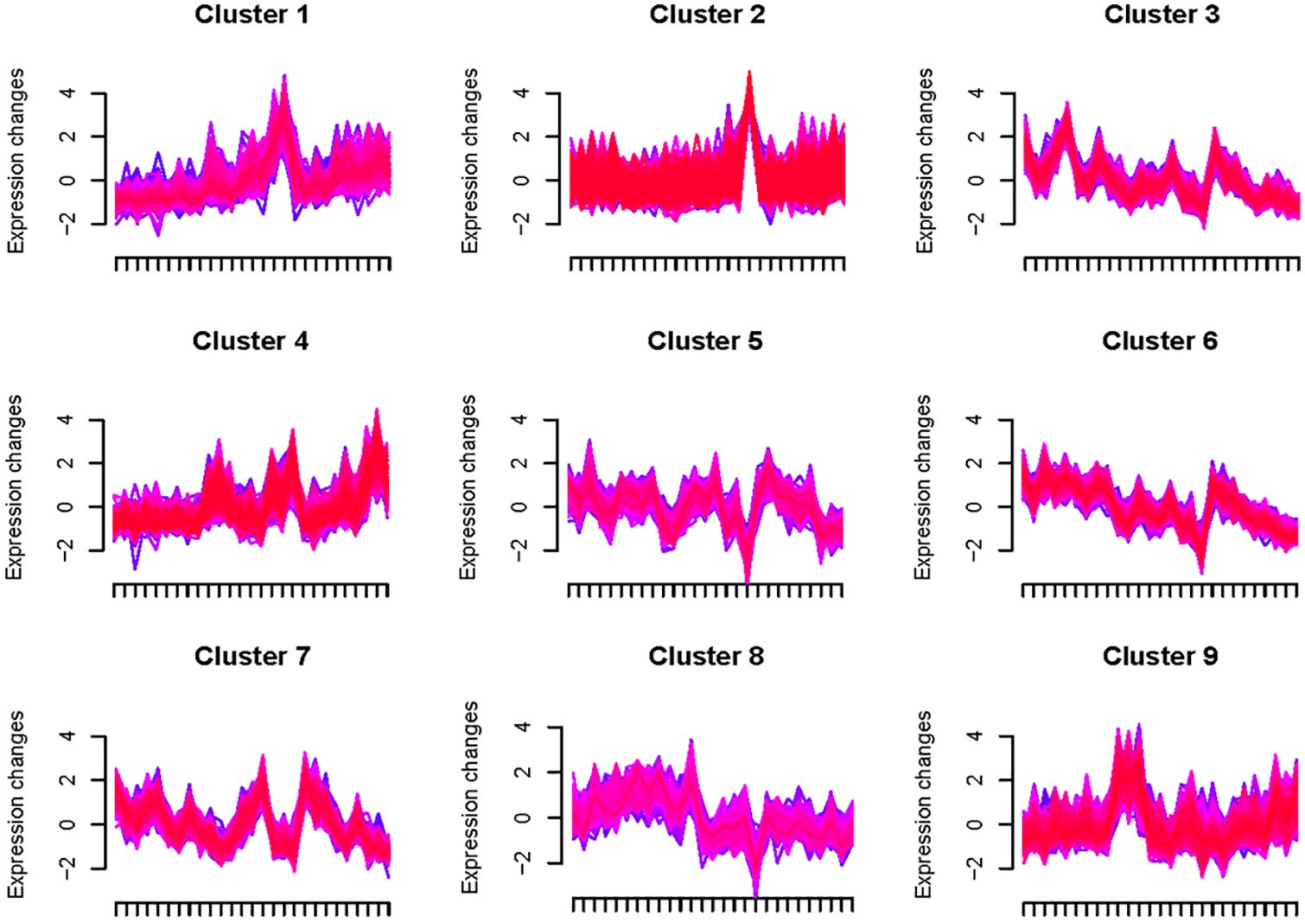
Nine gene clusters were discovered among different samples. Many genes could be classed into one cluster according to their similar expression patterns at different time points. X-axis represents different time points. Y-axis represents the expression value after homogenization.

As shown in Fig 3, the number of differentially expressed gene (DEGs) in sorghum florets increased along with treatment duration. For example, the number of DEGs in groups SorgCK_1-VS-SorgCK_3 and SorgLM_1-VS-SorgLM_3 were higher than those in groups SorgCK_1-VS-SorgCK_2 and SorgLM_1-VS-SorgLM_2, respectively. When the sorghum florets opened, DEGs were the most compared with the control, more than those in sorghum florets unopened. For example, the up- or down- regulated genes in SorgCK_1-VS-SorgCK_4 and SorgLM_1-VS-SorgLM_3 were 3,371 and 5,388, respectively. The number of DEGs increased along with the MeJA concentration within the same treatment time. For example, the number of DEGs in group SorgCK_1-VS-SorgHM_1 and SorgCK_2-VS-SorgHM_2 were higher than those in group SorgCK_1-VS-SorgLM_1 and SorgCK_2-VS-SorgLM_2, respectively. All results indicated so many DEGs were involved in the biological regulation of floret opening in sorghum. Besides, it was found that several genes may have similar expression patterns at different time points among different samples based on their expression quantity. So these genes could be clustered into one gene cluster which was correlated with floret opening based on their similar expression patterns. In our study, totally 9 gene clusters were discovered (Fig 4), suggesting that these genes followed certain expression rules at the opening stage of sorghum florets.

### Verification of DEGs using qRT-PCR

**Fig 5:**
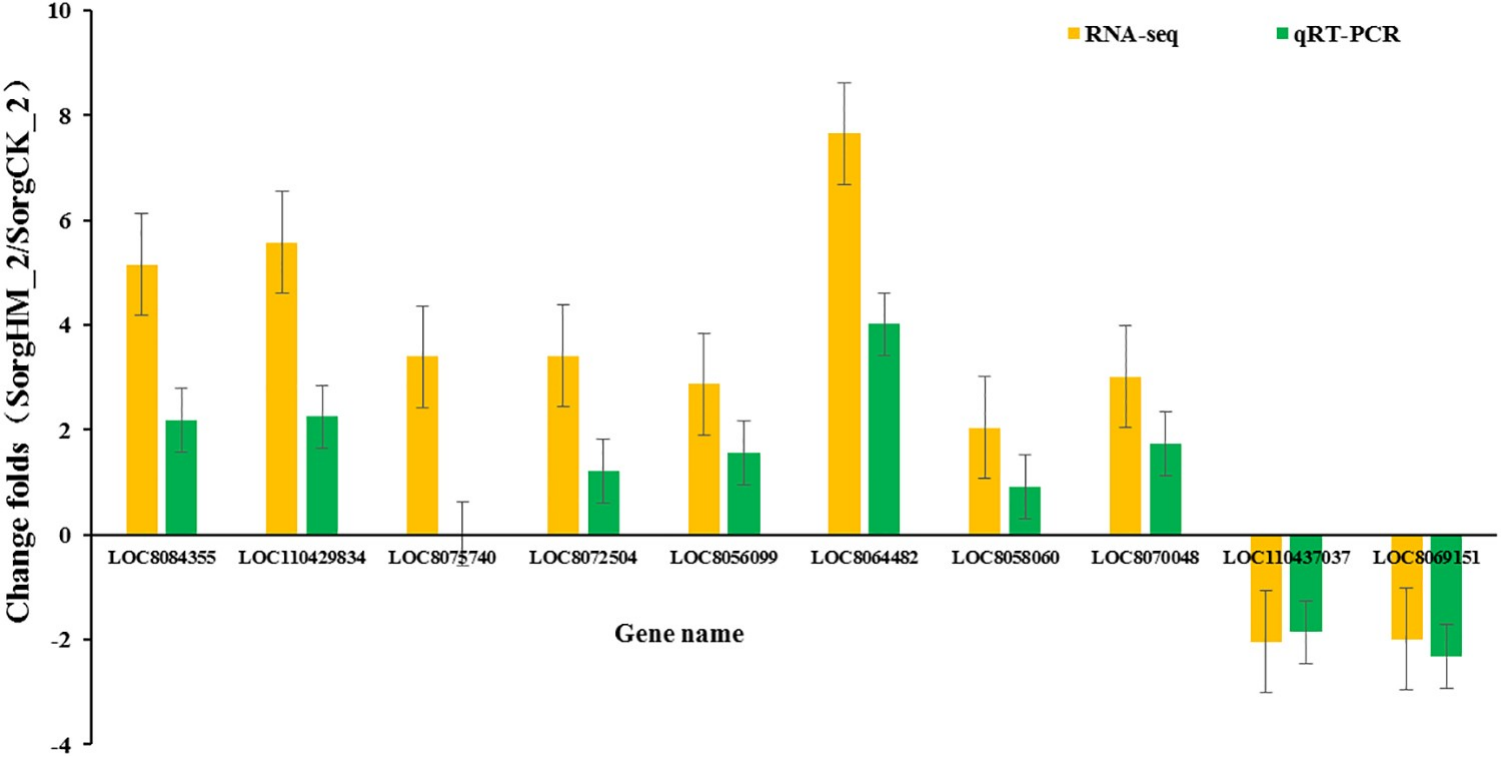
Comparison of the DEGs between qRT-PCR and RNA-seq. qRT-PCR was an effective method to verify gene expression changes originated from RNA-seq analysis. Although their relative expression values were not the same, the variation trends were consistent. Genes above the X-axis were up-regulated and the below genes were down-regulated.

To further verify the reliability of the transcriptome results, 17 genes with significantly different expression levels were selected (Table 2) for fluorescence quantitative RT-PCR to verify their expressions. Although the relative expression values determined by qRT-PCR did not exactly match the value from the transcriptional sequencing analysis, the variation trends of gene expression were the same at the given time points (Fig 5), which indicated the results of the RNA-seq analysis of gene expression were credible. Interestingly, 10 genes were obtained in several significant pathways by blast analysis in NCBI database. Among of which, *LOC8075740*, *LOC8056099*, *LOC8064482*, *LOC8058060*, *LOC110437037* and *LOC8069151* were involved in starch and sucrose metabolism, and LOC8084355 was involved in hormone signal transduction pathways and *LOC110429834* was involved in the α-linoleic acid metabolic pathway and *LOC8072504* was involved in the regulation of plant circadian rhythm.

**Table 2:** Genes and primers for DEG expression verification using qRT-PCR.

NO	Gene ID	Forward primer	Reverse primer
1	LOC8063704	GGCTACGCCTATATCCTCACG	AACGTAGAGATCGCCGTCAGC
2	LOC8083539	GTGCTGTGCTTGCTTATTGGC	TGCGCCTGGAGAAACTTGTAC
3	LOC8056206	GGCTACGCATACATCCTCACG	CTTTCTCCCAGACGGCATAAT
4	LOC8060604	AGGCAAGCTAGGCGTGGACA	CATTCTGAGAACCAGCTTCTGG
5	LOC8084355	GATGACCAACGGGTACCTCTC	TCCTGCTCCCTGTCCTTAATG
6	LOC110437037	TTTCCTGGAGCCAATGACTTT	TGGAGCAGAACGGTCATAGAA
7	LOC8069151	TGCTCGAATTGGGAAGAATGT	CCACAGTTATCCCAGAACGAAT
8	LOC8082558	GTGGTAGCAGGAGGCAGTAGC	ACAACCACGGGCGATTACAG
9	LOC8058060	GGCGAGTTCCAATGCTATGAC	CGTCAGGCATCTGCTTGTACTC
10	LOC8070048	CCTAACAGATATGCAATATGTGGTT	TGTGCCTAATGTAATAGTCCTGGT
11	LOC8075740	AGTGGGGCACCGACTACTTCAT	CTGCCAGCAGTAGTGGTCCGT
12	LOC8072504	ACTCTGTTAGCTTTCTACCTTTGC	AGATTCCAGTCAAGGCACAGC
13	LOC8064482	GTACCCTCCTGGTCACTGCTC	AACCACCTTGTTTCCCCTGAT
14	LOC110429834	TTACCTCTCGTTGACACCATC	CACACCACATCCTTGCTTTAG
15	LOC8056099	ATTTGGGACCAGGCAGACATT	GAGCACCAACAAACGCATCAC
16	LOC8083790	TAGCAAAAGATGTCAGTTTCAAGG	GTAATCCCATCGTATTCTTCCAAAA
17	LOC8155361	CCACTTCAAGTTCGGCCTCTT	AACCAGTGGGACTTCCAGACG

### Functional pathway analysis of DEGs

**Fig 6:**
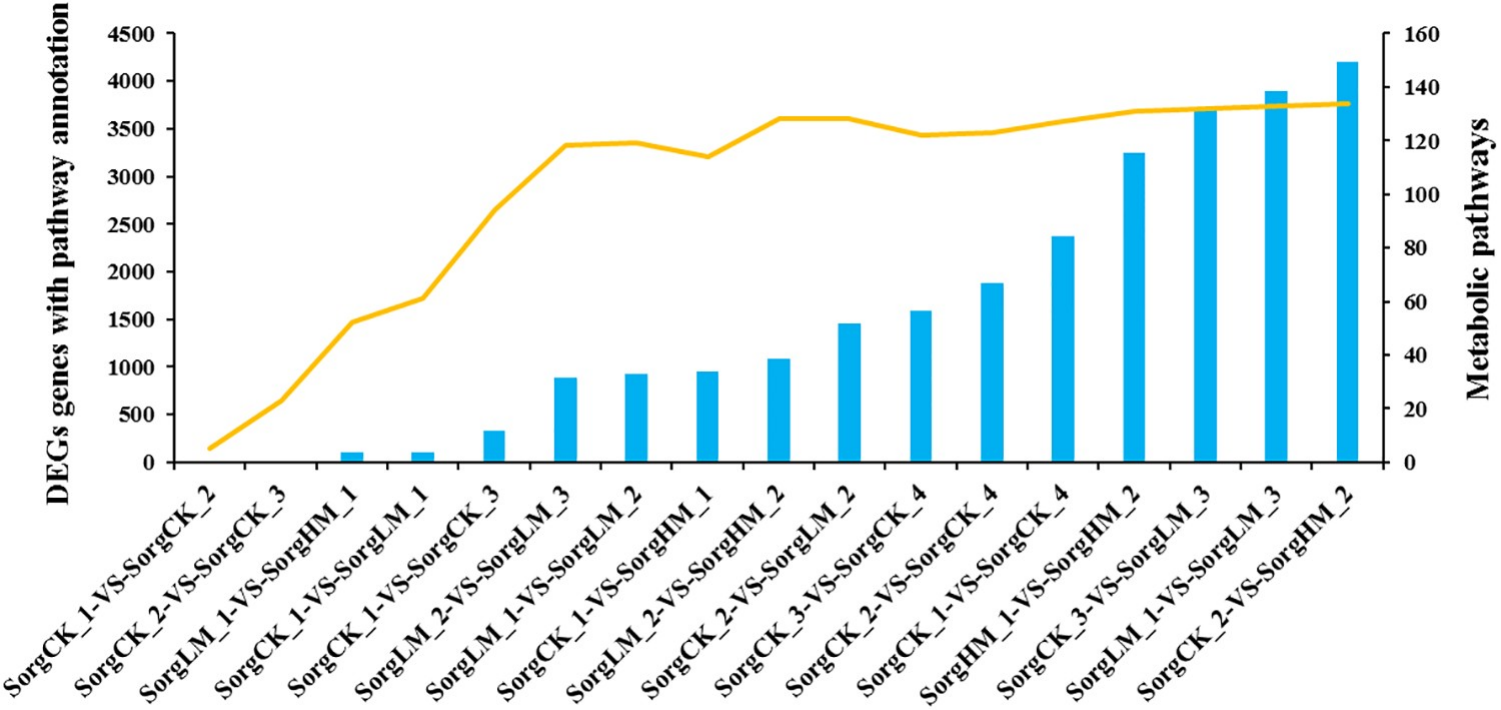
Functional analysis of differential expression gene pathway in sample contrast group. X-axis represents different VS groups. Y-axis represents the number of DEGS and metabolic pathways in each VS groups. SorgCK_1, SorgCK_2, SorgCK_3 and SorgCK_4 represented samples treated with 0 mmol/L MeJA at 19:00 (1h), 20:30 (2.5h), 22:30 (2.5h) and 0:30 (6.5h), respectively. SorgHM_1 and SorgHM_2 represented samples treated with 2.0 mmol/L MeJA at 19:00 (1h) and 20:30 (2.5h), respectively. SorgLM_1, SorgLM_2 and SorgLM_3 represented samples treated with 0.5 mmol/L MeJA at 19:00 (1h), 20:30 (2.5h) and 22:30 (4.5h), respectively.

Based on the results of DEGs detection, biological pathway classification and enrichment analysis was performed in KEGG. Total of 29,135 unigenes were used to blast in the KEGG database, and finally 20,803 (70.96%) unigenes were annotated (Fig 6). In the 17 control groups, group SorgCK_1-VS-SorgCK_2 enriched the least metabolic pathways while group SorgCK_2-VS-SorgHM_2 enriched the most metabolic pathways (134). In the group SorgCK_2-VS-SorgHM_2, 4,198 DEGs were totally annotated. The number of metabolic pathways and annotated genes increased with prolonged treatment duration under the same treatment of exogenous MeJA in sorghum. For example, the number of enriched metabolic pathways and annotated DEGs in group SorgCK_2-VS-SorgHM_2 were significantly greater than that in group SorgCK_1-VS-SorgHM_1. Besides, the number of enriched metabolic pathways and annotated DEGs in group SorgCK_3-VS-SorgLM_3 were obviously greater than that in group SorgCK_2-VS-SorgLM_2 and SorgCK_1-VS-SorgLM_1.

At the same time point, the number of enriched metabolic pathways and DEGs increased along with the increase of MeJA concentration. For example, the number of enriched metabolic pathways and DEGs in SorgCK_1-VS-SorgHM_1 were greater than that in group SorgCK_1-VS-SorgLM_1. And the number of enriched metabolic pathways and DEGs in group SorgCK_2-VS-SorgHM_2 was greater than that in group SorgCK_2-VS-SorgLM_2.

In order to dig out the pathways which were most closely associated with spikelets opening, screening was performed with the following rules: 1) total number of DEGs > 3,000; 2) number of enriched metabolic pathways > 100; and 3) Q value < 0.05. Finally four comparison groups met the criteria, including SorgCK_3-VS-SorgLM_3, SorgCK_2-VS-SorgHM_2, SorgLM_1-VS-SorgLM_3 and SorgLM_3-VS-SorgLM_3. Totally nine metabolic pathways were enriched, including alpha-Linolenic acid metabolism, glycerolipid metabolism, glycerophospholipid metabolism, plant hormone signal transduction, anthocyanin biosynthesis, phenylpropanoid biosynthesis, starch and sucrose metabolism, limonene and pinene degradation, and circadian rhythm—plant (Table 3).

**Table 3:** Numbers of metabolic pathways enriched with different genes from several sample groups.

Groups	SorgCK_2-VS-SorgHM_2 (4198)	SorgLM_1-VS-SorgLM_3 (3892)	SorgCK_3-VS-SorgLM_3 (3717)	SorgHM_1-VS-SorgHM_2 (3250)
Metabolic pathways				
alpha-Linolenic acid metabolism	44	0	37	26
Glycerolipid metabolism	84	68	71	65
Glycerin metabolism	50	48	60	43
Plant hormone signal transduction	192	186	190	127
Anthocyanin biosynthesis	18	16	18	0
Phenylpropanoid biosynthesis	136	0	133	112
Starch and sucrose metabolism	130	0	117	107
Limonene and pinene egradation	17	0	14	0
Circadian rhythm—plant	0	55	49	40

### Analysis of DEGs in JA related pathway

**Fig 7:**
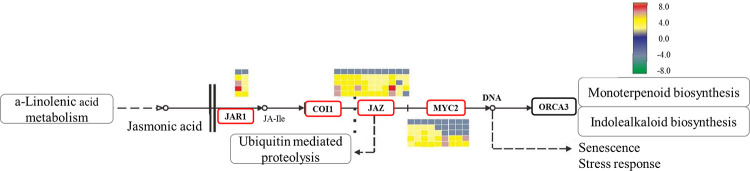
Detailed information for the JA signal transduction pathway. JAR1: Jasmonate amino acid-binding enzyme, COI1: Coronatoxin insensitive protein 1; JAZ: Jasmonate ZIM domain protein, MYC2: MYC2 transcription factor, ORCA2/3: AP2 transcription factor DNA-binding protein.

JA and isoleucine (Ile) could form a conjugate (JAalle) when catalyzed by jasmonic acid-amido synthetase (JAR1), which induces the binding of the Jas motif in the jasmonate ZIM domain and COI1. The JAZ motif was then degraded, which was followed by the dissociation of the NINJA-TPL and transcription factor MYC2, resulting in the transcription of JA signaling-related genes [32–34]. On the basis of the transcriptome analysis, 19 DEGs were discovered at the same time point from four control groups (excluding SorgCK_1-VS-SorgLM_1) in JA signal transduction pathways (Fig 7), including two JAR1-related genes (*LOC8084842* and *LOC8065295*) that catalyze the binding of JA and Ile. The expression level of *LOC8084842* significantly increased after the treatment with 2.0 mmol/L MeJA for 2 hr, and the score reached to 7.6. In addition, 10 genes, including *OsJAZ13* (*LOC8067868*, *LOC8067866* and *LOC8067865*), *OsJAZ10* (*LOC8083641*, *LOC8062786* and *LOC8072010*), OsJAZ8 (*LOC8062740*), *OsJAZ2* (*LOC8079184*), *OsJAZ9* (*LOC8059788*) and *OsJAZ6* (*LOC8077075*), were found to be correlated with the JAZ protease activity. These genes could promote the degradation of JAZ, and significant differences were observed after the treatments of 2.0 mmol/L MeJA for 2 hr and 0.5 mmol/L for 4.5 hr compared with the controls. The difference score for LOC8072010 was 6.4. Seven genes, including *LOC8069641*, *LOC8057408*, *LOC110436805*, *LOC8055721*, *LOC110433436*, *LOC8059158* and *LOC8060491*, were found to be correlated with bHLH zip transcription factors. These genes promoted MYC2 transcription initiation and the difference score reached 2.0 compared with the controls when treated with 2.0 mmol/L MeJA for 2 hr and 0.5 mmol/L MeJA for 4.5 hr. All results indicated that exogenous MeJA could induce the differential expression of these important genes, including *JAR1*, *JAZM* and *MYC2*. This demonstrated the important regulatory function of exogenous MeJA in sorghum floret opening at the molecular level.

The JAs are a class of fatty acid derivatives which play important regulatory roles in plant growth and development, mechanical damage and the induction of defense-related gene expression. JAs are mainly biosynthesized through the α-linolenic acid metabolic pathway. Lecithin on the cell membrane is catalyzed to release the precursor linolenic acid for the synthesis of JA, which is then oxidized to 13S-hydroperoxylinolenic acid through catalysis by lipoxygenase (LOX, EC 1.13.11.12). 13S-Hydroperoxylinolenic acid subsequently forms 12S,13S-epoxylinolenic acid, followed by cyclization to form 12-oxo-phyto-dienoicacid, which is then subject to be catalyzed to form MeJA or other complexes/metabolites [35]. As shown in S1 Fig, except for the group SorgCK_1-VS-SorgLM_1, 22 DEGs in the five control groups were involved in the JA biosynthetic pathway, including one gene related to the activity of lecithin lipid A1 (EC: 3.1.1.32) (*LOC8085653*) and four genes related to the activity of lecithin A2 (EC: 3.1.1.4) (*LOC8070964*, *LOC8057399*, *LOC8079812* and *LOC8077453*). The differential expression score of *LOC8085653* in SorgCK_2-VS-SorgHM_2 was 5.4, which was the most remarkable differential expression among the five groups. Besides, three genes related with lipoxygenase activity were also found, including *LOC8082015*, *LOC8055177* and *LOC8065835*. All these genes were up-regulated in the α-linolenic acid metabolic pathway, positively regulating the biosynthesis of JA.

### Two genes named *LOC*-1 and *LOC-2* played an important role in the opening process of sorghum florets

**Fig 8:**
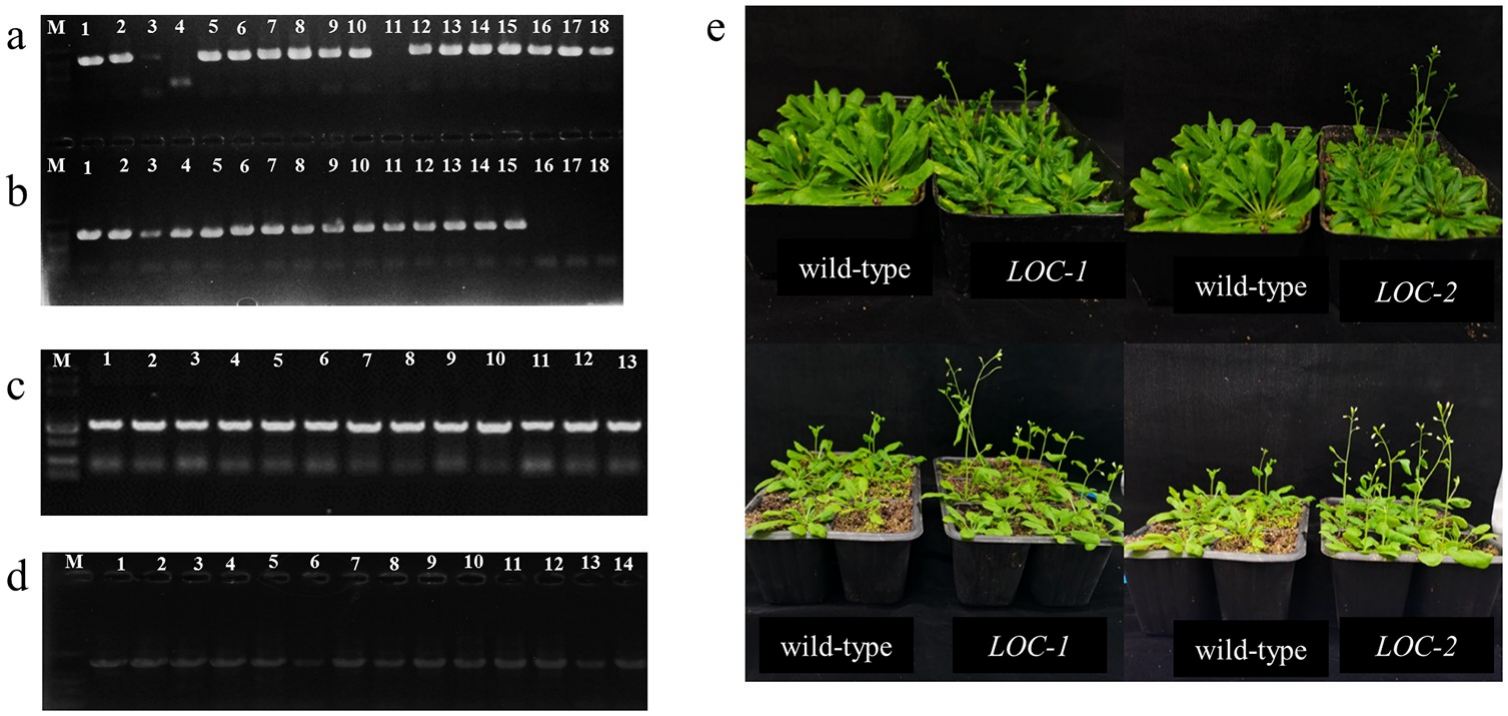
Functional identification of two targets *LOC-1* and *LOC-2*. a, Gene cloning of target *LOC-1*. Lines 1–8 were the PCR results of *LOC-1*. b, Gene cloning of target *LOC-2*. Lines 1and 3 were the PCR results of *LOC-2*. c, PCR identification of positive plants of *LOC-1*. Lines 1–13 represent 13 positive plants of *Arabidopsis thaliana* by PCR. d, PCR identification of positive plants of *LOC-2*. e, Comparison of flowering time in *Arabidopsis thaliana*.

In addition, another pathway named starch and sucrose metabolic pathway was found so important in the process of sorghum floret opening. As is well-known, starch and sucrose metabolism is a complex process of sugar metabolism. Starch synthesis starts from the degradation of sucrose by sucrose synthase (EC: 2.4.1.13) into uridine diphosphate glucose (UDPG) and fructose, followed by a series of biochemical reactions to form starch [36]. AGPase is the first enzyme in the process of plant starch biosynthesis, as well as being the rate-limiting enzyme of the whole process. The analysis in the study showed that *LOC110437037*, *LOC8069151*, *LOC8064663* and *LOC8060513* were related to the AGPase activity and five genes *LOC8066439*, *LOC8081295*, *LOC8066807*, *LOC8068976* and *LOC8071331* were related to GBSS and GBE activity levels (S2 Fig). Expression analysis showed that these genes were downregulated at varying degrees. On the other hand, two enzymes named AMY and BMY (*LOC8063704*, *LOC8083539*, *LOC8056206* and *LOC8072062*) were significantly upregulated to promote the degradation of starch. Two target genes named *LOC-1*, playing an important role in the starch and sucrose metabolic pathway, and *LOC-2*, playing an important role in energy metabolic pathway, were selected and cloned, then transformed into wild-type *Arabidopsis thaliana* (S1 Table and Fig 8a–8d). The results indicated the transgenic plants started to flower on the 30^th^ day, while the wild-type *A*. *thaliana* delayed to flower by 7 days. The statistical results of the opening spikelets showed the wild-type *A*. *thaliana* does not begin to produce its first flower until the transgenic *A*. *thaliana* has reached 30 or more flowers, indicating overexpression of two genes could induce the earlier flowering of transgenic plants (Fig 8e). In summary, sorghum floret opening was consistent with the reduction of starch biosynthesis in cells accompanied by an accelerated metabolism. Therefore, the starch level decreased in lodicules cells, degrading into other sugars to reduce the osmotic pressure in the cells, which was correlated with the earlier opening of florets in sorghum.

## Discussion

Sorghum is a short-day tropical species with the characters of substantial photoperiod sensitivity and delayed flowering in long days [37]. Sorghum genotypes show a wide-range of photoperiod sensitivity and historic genetic studies uncovered six flowering-time loci, namely *M*_*a1*_, *M*_*a2*_, *M*_*a3*_, *M*_*a4*_, *M*_*a5*_ and *M*_*a6*_ [38,39], but less literatures about the molecular mechanism of sorghum florets opening regulated by exogenous MeJA. Literatures reported that MeJA or JA treatments could significantly induce rice floret open [12]. Documents also reported that qRT-PCR method could be used to study the dynamic expression levels of JA signal-related genes during rice floret opening and showed that the expression levels of JA biosynthetic- related genes *OsDAD1*, *OSAOS1*, *OSAOC* and *OSOPR7*, signal transduction related genes *OsJAR1* and *OsCOI1b*, and 13 genes of the OsJAZ family, except *OsJAZ5* and *OsJAZ15*, in lodicules cells were significantly up-regulated compared with the expressions at 18 h before floret open [39]. This further demonstrated the involvement of endogenous JA in the regulation of floret-opening in rice.

In this study, RNA-seq method was used to explore transcriptome changes in lodicule cells during sorghum floret opening after exogenous MeJA treatments. 0.5 and 2.0 mmol/L MeJA were applied to treat sorghum florets and the results showed the number of DEGs annotated and functionally metabolic pathways increased along with the increase of MeJA concentration. Functional enrichment analysis of DEGS showed plant hormone signal transduction and starch and sucrose metabolism were closely related with the open of sorghum florets. Thus, the results in this study showed MeJA could induce various physiological metabolisms in lodicules of sorghum florets, thereby promoting the floret open in advance. The results of this study are consistent with the previous reports, further confirming the regulatory mechanism we proposed in gramineal crops.

JA biosynthesis begins with the release of the precursor α-linolenic acid from lecithin on the cell membrane, followed by a cascade of reactions catalyzed by multiple enzymes, including *DAD1*, *LOX*, *AOS*, *AOC* and *OPR3*. As shown in S1 Fig, differentially expressed genes (DEGs) identified under the treatment of 0.5 mmol/L MeJA at three time points were 2, 19 and 30 compared with the control, respectively. Differentially expressed genes (DEGs) identified under the treatment of 2.0 mmol/L MeJA were 10 and 37 at two time points compared with the control, respectively. This result showed that 2.0 mmol/L MeJA could induce more DEGs than 0.5 mmol/L MeJA at the same time point. Taking log_2_ratio = 3.0 as the criterion, significant expression differences were observed under the high concentration of 2.0 mmol/L treatment, including DAD1 activity related genes *LOC8057399* (log_2_ratio = 3.5), *LOC8085653* (log_2_ratio = 5.4) and *LOC8055636* (log_2_ratio = 4.2), AOS activity related genes *LOC8056861* (log_2_ratio = 3.1), 12-oxo-dienoic acid reductase related genes *LOC8070772* (log_2_ratio = 3.9) and *LOC8070773* (log_2_ratio = 3.3), OPC-8:0 coenzyme A ligase related gene *LOC8063048* (log_2_ratio = 5.0). Interestingly, 19 genes including *LOC8057399*, *LOC8082061*, *LOC80770776* and *LOC8068239* and etc., were significantly upregulated under the treatment of 2.0 mmol/L, while no expressions were found under the treatment of 0.5 mmol/L. These 19 genes were actively involved in the biosynthesis of JA, and high expression levels of these genes could further increase the content of JA, which was favorable to the open of sorghum florets.

The JA signal transduction pathway starts with the JAZ complex, followed by enzyme catalysis by JAR1, JAZ and MYC2 to induce JA signals. As shown in Fig 7, it was also found that several genes, including *LOC8062786*, *LOC8083641*, *LOC8059386*, *LOC8056732*, *LOC110436805*, *LOC110429687*, *LOC8055721*, *LOC8055143*, *LOC8068250* and *LOC110430094* were only differentially expressed under the 2.0 mmol/L MeJA treatment and these genes were barely expressed under the 0.5 mmol/L MeJA treatment, which suggested that 2.0 mmol/L MeJA treatment induced the expression of these genes to promote the open of sorghum florets by JA signal transduction.

In this study, four genes correlated with AGPase, which is a rate-limiting enzyme in starch synthesis and metabolism, were all downregulated, while four genes correlated with AMY and AMS were significantly upregulated to promote the degradation of starch. This result suggested that exogenous MeJA induced the up-regulation of starch synthesis related genes and the down-regulation of starch degradation related genes, thereby promoting the degradation of starch in lodicules cells into sucrose, maltose or other sugars, which caused the decrease of the osmotic potentials in lodicule cells. This further allowed lodicule cells to absorb water and swell, resulting in floret open. Previous research reported that the altered expression patterns of genes involved in glycolysis/gluconeogenesis pathway could cause early flowering in rice [40,41]. In addition, two important genes named *LOC-1* and *LOC-2* were cloned and transformed into *Arabidopsis thaliana*, overexpression of these two genes resulted the earlier flowering in transgenic *Arabidopsis* plants. This was not reported before.

**Fig 9:**
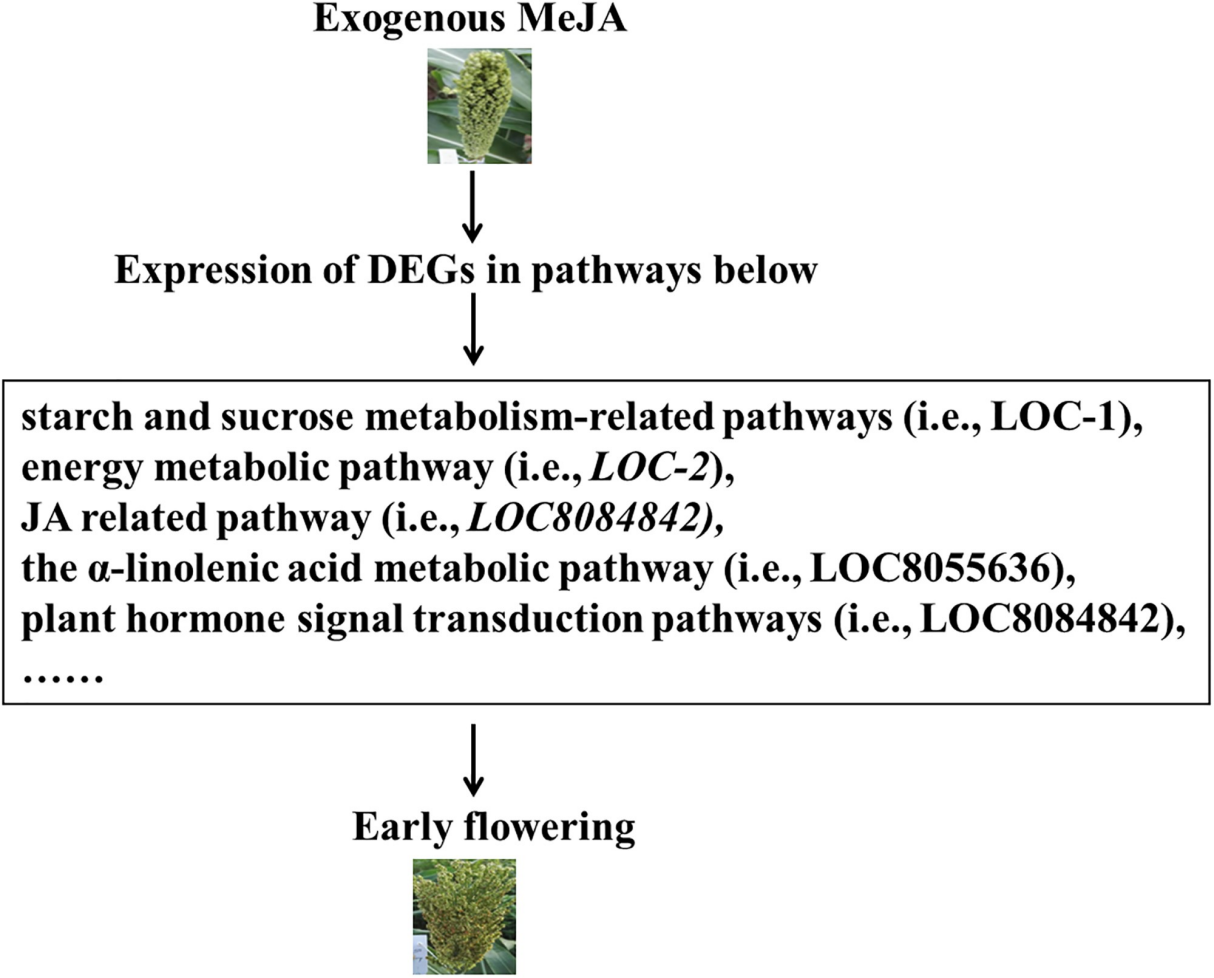
A finally proposed model involved in the florets opening mechanism.

In conclusion, floret opening is initiated by water absorption and lodicule swelling that pushes the chaff out. This process requires the loosening of lodicule cell walls and the reduction of the solute potential in lodicule cells. The results of this study showed that exogenous MeJA could promote the degradation of starch and the reduction of the solute potential in sorghum lodicule cells at the molecular level (Fig 9), but the detailed molecular mechanism that promotes starch degradation and its possible impact on the loosening of lodicule cell walls still requires further investigation.

## Supporting information

S1 Fig Pathway analysis of linolenic acid metabolism. (TIF)

S2 Fig Pathway analysis of starch and sucrose metabolism. (TIF)

S1 Table Full information of two targets *LOC-1* and *LOC-2*. (XLSX)
