# Supplementary figures and images for: Transcriptome analysis of the impact of exogenous methyl jasmonate on the opening of sorghum florets
Source: PLoS One. 2021 Mar 31;16(3):e0248962. doi: 10.1371/journal.pone.0248962 (PMC8011725; doi:10.1371/journal.pone.0248962)

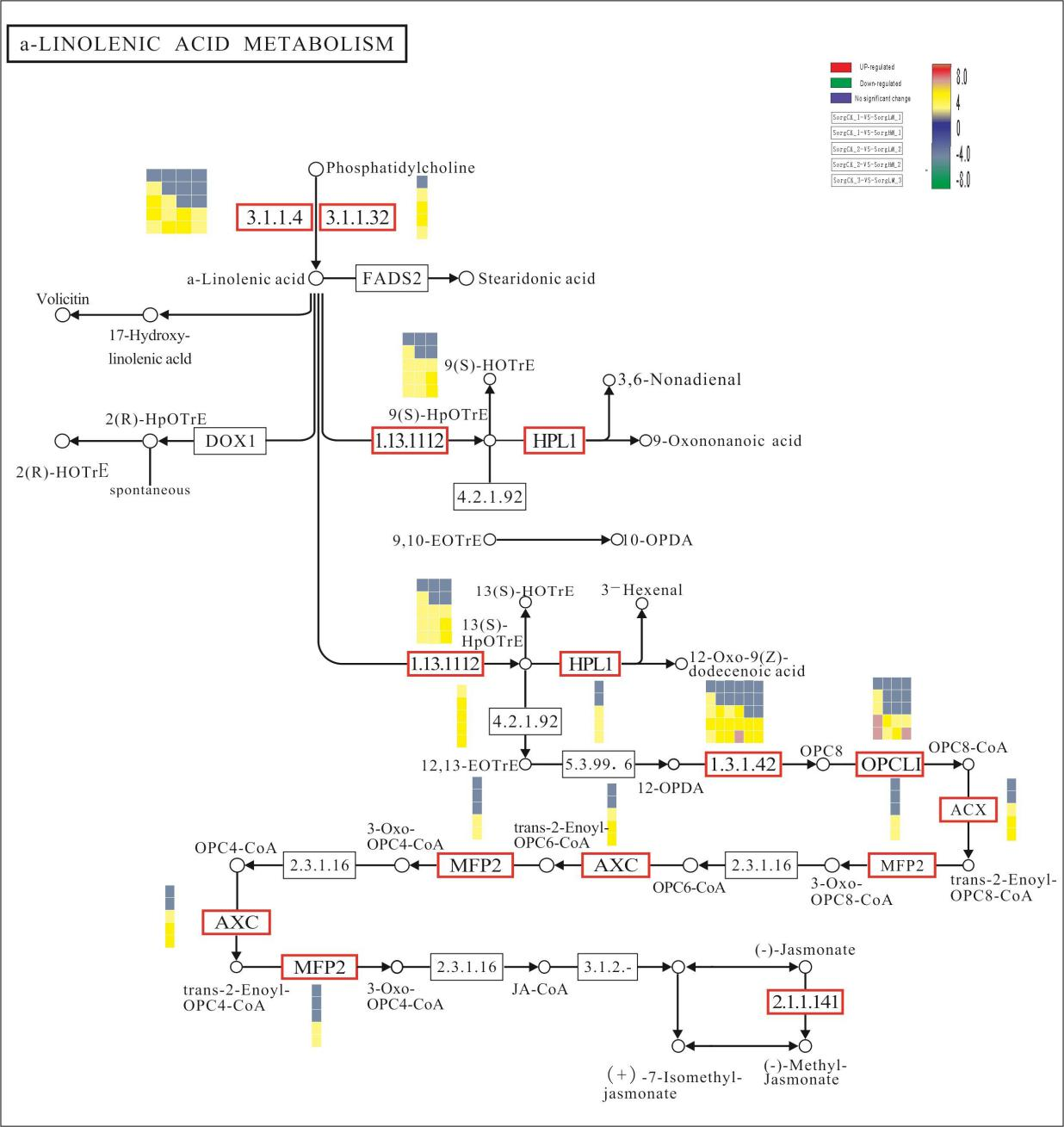

Supplement: S1 Fig — (TIF) [file pone.0248962.s001.tif]

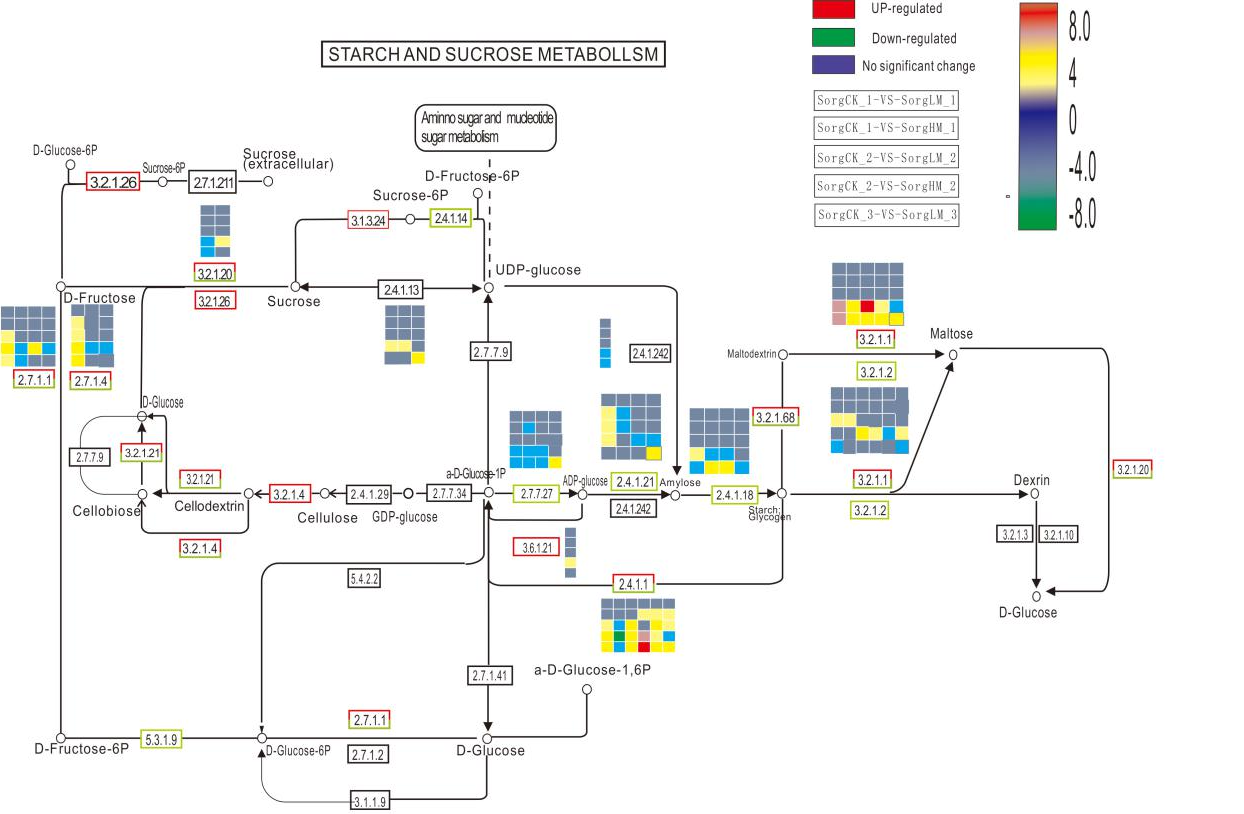

Supplement: S2 Fig — (TIF) [file pone.0248962.s002.tif]
